# Quantification of the Spatial Organization of the Nuclear Lamina as a Tool for Cell Classification

**DOI:** 10.1155/2013/374385

**Published:** 2013-11-07

**Authors:** Christiaan H. Righolt, Diana A. Zatreanu, Vered Raz

**Affiliations:** ^1^Manitoba Institute of Cell Biology, CancerCare Manitoba, University of Manitoba, 675 McDermot Avenue, Winnipeg, MB, Canada R3E 0V9; ^2^Department of Imaging Science & Technology, Delft University of Technology, Lorentzweg 1, 2628 CJ Delft, The Netherlands; ^3^Leiden University Medical Center, Department of Human Genetics, P.O. Box 9600, 2300 RC Leiden, The Netherlands

## Abstract

The nuclear lamina is the structural scaffold of the nuclear envelope that plays multiple regulatory roles in chromatin organization and gene expression as well as a structural role in nuclear stability. The lamina proteins, also referred to as lamins, determine nuclear lamina organization and define the nuclear shape and the structural integrity of the cell nucleus. In addition, lamins are connected with both nuclear and cytoplasmic structures forming a dynamic cellular structure whose shape changes upon external and internal signals. When bound to the nuclear lamina, the lamins are mobile, have an impact on the nuclear envelop structure, and may induce changes in their regulatory functions. Changes in the nuclear lamina shape cause changes in cellular functions. A quantitative description of these structural changes could provide an unbiased description of changes in cellular function. In this review, we describe how changes in the nuclear lamina can be measured from three-dimensional images of lamins at the nuclear envelope, and we discuss how structural changes of the nuclear lamina can be used for cell classification.

## 1. Composition of the Nuclear Lamina

The nuclear envelope (NE) is a biostructure, which separates the nuclear and cytoplasmic parts of eukaryotic cells. The NE is a dynamic structure composed of the outer nuclear membrane (ONM) and the inner nuclear membrane (INM). The NE is embedded with nuclear pore proteins, through which molecules selectively pass to move between the nucleus and the cytoplasm. The nuclear membranes are bilipid structures supported by a network of proteins. Within the nucleus, the INM is underlined by the nuclear lamina, a dynamic filamentous protein network. The nuclear lamina has both regulatory and structural roles.

The nuclear lamina is predominantly composed of lamins, which are dynamically anchored to the INM via posttranslational modifications. The molecular composition of the nuclear lamina has been previously discussed in several reviews [1, 2]. In brief, lamins are divided into A- and B-types (LMNA and LMNB, resp.) and play a central role in the integrity of the nuclear lamina. Both proteins bind to the chromatin at highly defined regions, creating a regulatory role for lamina-chromatic interaction [3]. While *LMNB* is constitutively expressed, the expression of *LMNA* is developmentally regulated, and expression levels differ between cell types. It has, therefore, been suggested that LMNA also plays a regulatory role [1, 2]. Silencing of *LMNB* causes dramatic changes in the LMNA meshwork, while *LMNA* downregulation has no effect on the structural organization of *LMNB* filaments [1]. This suggests a crucial and indispensable role for LMNB in maintaining the structural integrity of the nuclear lamina. 

Lamins are brought into the INM via a farnesyl group, which is attached to the C-terminus of the molecule by a posttranslational modification. The maturation step of the B-type lamins ends with a farnesylated molecule, while the mature LMNA protein is generated after a reversible anchorage to the INM via the farnesylated group [4]. Reversible LMNA farnesylation is essential for proper maintenance of nuclear shape. In progerin, a *de novo* mutation in the last intron-exon boundary in LMNA causes removal of the 3′ of exon 11 and a deletion of the C-terminus, including the farnesylation signal. As a result, farnesylation is irreversible, and the progerin protein predominantly attaches to the IMN. The nuclear shape in progerin expressing cells is consequently severely deformed [5]. *In vitro*, progerin causes premature cell senescence, which is associated with genome-wide transcriptional changes and telomere shortening [6, 7]. Progerin is the genetic cause for Hutchinson Gilford Progeria Syndrome (HGPS). Progeria patients exhibit premature aging, starting in young children, only a few years old [8]. Progerin is also expressed during normal aging [6, 9] and during closure of the *ductus arteriosus* in neonates [10]. In the closing *ductus arteriosus*, progerin is expressed in cells with increased apoptosis. The function of progerin in those cells is still obscure, and the mechanism regulating progerin expression in normal physiological situations remains to be studied. 

## 2. Conditions with Deformed Nuclear Lamina

Next to genetic conditions, a deformed nuclear structure is also found in senescent, aging, and apoptotic cells as well as during cell division [11–13]. Moreover, overexpression of LMNA in cell culture also causes severe changes in the nuclear shape [14]. In those cells, folding of the nuclear lamina is associated with uneven distribution and local accumulation of lamin A [15]. The molecular mechanisms driving the changes in the nuclear lamina in senescent and aging cells are not fully understood. The changes in the nuclear lamina during cell division have, however, been studied extensively and are described in several reviews [1, 4, 16]. In this review, we will focus on the structural changes of the nuclear lamina that are associated with cell senescence and aging.

In apoptotic cells, the nuclear envelope is irreversibly degraded by the apoptosis-regulated caspases [17]. Changes in the structure of the nuclear lamina in human mesenchymal stem cells (hMSCs) precede DNA fragmentation, the hallmark of cell apoptosis. Cleavage of lamins by caspase-3 follows structural deformation of the nuclear lamina structure [11]. This suggests that structural deformation of the nuclear lamina precedes irreversible degradation of lamins during caspase-8 induced apoptosis. Changes in the shape of the nuclear lamina also precede well-known bioimaging markers for cell senescence [11, 13, 17]. This suggests that monitoring changes in the nuclear lamina organization could mark early changes in cellular function. An unbiased and robust methodology is required to achieve this.

## 3. Detection of the Nuclear Lamina

The nuclear lamina is a three-dimensional structure. Architectural changes should, therefore, be investigated in living cells expressing lamins fused to fluorescent proteins [14]. As high overexpression of lamins leads to structural deformation of the nuclear lamina, only cells with low expression level should be considered for analysis of the lamina structure. The structure is determined from three-dimensional images. To avoid overexpression, analysis can be also performed on three-dimensional reconstructions from immunofluorescence. Those are, however, less robust compared with those obtained from living cells [18]. The pixels associated with the nuclear lamina are then identified using automated image processing procedures. Several studies reported the quantification of the shape of the cell nucleus in two dimensions [19, 20]. Those analyses only account for the edge of the nucleus and do not provide an internal architectural description. Recently, we developed an automatic method to detect pixels of the nuclear lamina from three-dimensional images. Quantitative analysis of the lamina architecture was performed subsequently, resulting in a robust and reliable procedure to study cell populations [18].

## 4. Biophysical Properties of the Nuclear Lamina in Aging Cells

**Figure 1:**
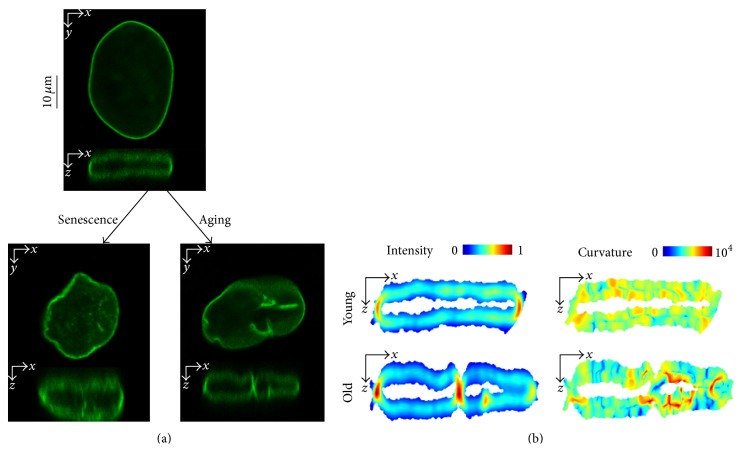
(a) Confocal microscopy images of living cells expressing LMNA fused to GFP in human mesenchymal stem cells (cross-sections are shown). LMNA-GFP transgene was expressed in cells by a lentivirus expression system. Spatial changes in the structure of the nuclear lamina are shown in a lateral (*x,y*)-plane and in a (*x,z*)-plane. The top shows a typical nucleus of an early passage from a 35-year-old donor. Bottom left shows a nucleus from the same donor but at a late passage number. This exemplifies how the nuclear lamina structure changes in senescent cells. Bottom right panel depicts a typical nucleus of an early passage cell from an 81-year-old donor. (b) LMNA is redistributed at the nuclear lamina concurrent with the change in the shape. The “even distribution” at the “short edges” of the structure in young cells (upper left) becomes more heterogeneous along the nuclear lamina for the older cell (bottom left). Protein accumulation is illustrated with red for high values and blue for low values; the color scale is linear. The curvature of the lamina locally increases along the structure (lower right) compared to the young cell (upper right). Red indicates high curvature values and blue low values; the scale is logarithmic. The sections shown match the cross-sections in (a).

The nuclear shape can be described by curvature measures. In conditions where cells exhibit a deformed nuclear shape, as in senescent cells or disease situations, an increase in folding of the lamina occurs. Folding of the nuclear lamina is associated with high curvature values and often shows high fluorescence intensity values of LMNA, indicating protein accumulation. In young and healthy cells, LMNA is evenly distributed along the nuclear rim, and it accumulates mainly in the “short edges” of the nuclear surface. In aging or senescent cells, the main changes in the structure of the nuclear lamina are associated with an uneven distribution of intensity of LMNA and folding along the structure (examples are depicted in Figure 1(b)). Regions with high intensity values are often surrounded by high curvature values (Figure 1(b)), suggesting a biomechanical association where protein accumulation induces a mechanical pressure that causes bending of the structure. Changes in the nuclear lamina could be accurately reported when both features were quantified.

Several approaches can be used to quantify the three-dimensional structure of the nuclear lamina. A simple method is quantifying the length-to-width ratio of nuclear objects. Structural changes along the lamina would then not be detected, because only the overall length and width of the cell are measured. A more accurate description can be provided by curvature measures of the folding of the structure [18]. The distribution of fluorescence intensity at the nuclear lamina can provide a quantitative description of the structure as well. The intensity distribution can be summarized by the mean value and the skewness of the intensity distribution [15]. Absolute curvature and intensity values cannot be directly compared between cells; the measured values should be normalized first. A cell-based accurate and robust description of the nuclear lamina can then be obtained by combining these features [18]. With a linear classification method it is possible to determine whether cell populations can be classified based on the nuclear lamina structure. A comparison between different cell populations is possible, because the measures themselves are normalized [15]. The result of this robust analysis demonstrated that young and aged cell populations can be discriminated based on mean intensity and curvature values of the nuclear lamina (Figure 3).

## 5. Formation of Folds

**Figure 2:**
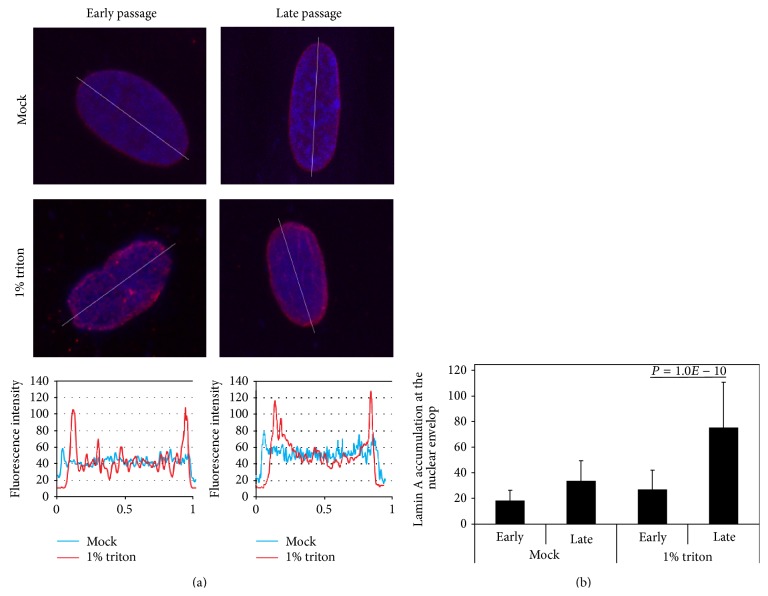
LMNA accumulation on the nuclear envelop is enriched in senescent cells. (a) Images of representative nuclei show LMNA staining in WI38 fibroblasts at early and late passage number (passage 13 and 27, resp.). Cells were mock treated or treated with 1% triton prior to fixation. LMNA is visualized with mouse-antibodies in red, and the DNA is counterstained with DAPI. After triton treatment, heterochromatic foci are disrupted. LMNA fluorescent intensities over the lines through the nuclei are shown in the lower figures. (b) LMNA enrichment at the nuclear envelope. LMNA enrichment at the nuclear envelop is normalized to the LMNA signal across the nucleus. Means and standard deviations are shown for 100 nuclei.

**Figure 3:**
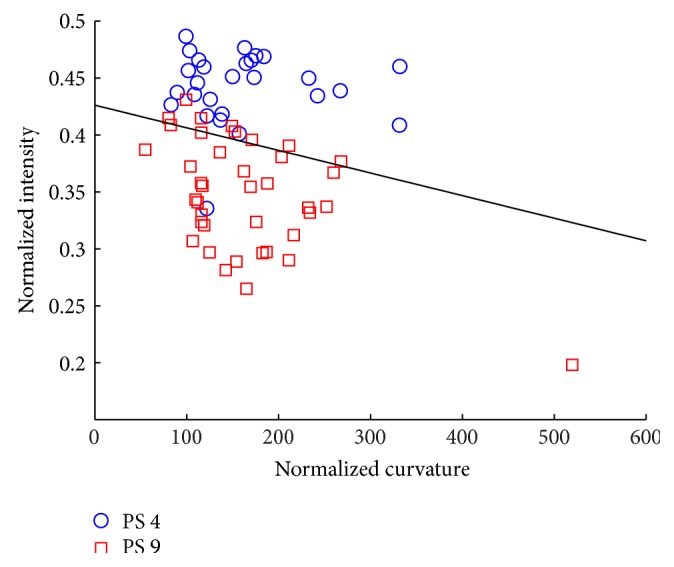
Cell classification of human mesenchymal stem cells at passage 4 (PS 4; blue circles) and passage 9 (PS 9; red squares) based on the normalized curvature and intensity. A linear classifier—the black line in the scatter plot—separates the two cell types with an error rate of 13% (see [18] for technical details). These features can be used to discriminate between the younger and older cells.

A confined accumulation of proteins is often a result of a decrease in protein mobility. Protein mobility can be measured by fluorescent recovery after photobleaching (FRAP) [21]. Using FRAP analyses, LMNA at the nuclear envelop is more mobile compared to LMNB [19]. In addition, the mobility of progerin is significantly reduced compared to the wild type LMNA protein [22, 23]. Triton treatment of cells disrupts the localization of soluble proteins, and only insoluble proteins and structures that are Triton-resistant remain intact. In cells at low passage number, LMNA enrichment at the nuclear envelope is significantly lower compared with cells at high passage number (Figure 3). The enrichment in LMNA accumulation at the nuclear envelope is significantly more pronounced after Triton treatment (Figure 2). This procedure suggests that LMNA at the nuclear envelope is less mobile in senescent cells than in young cells. When zooming into the nuclear envelop with the FRAP procedure, lower LMNA mobility was found at regions with high protein accumulation compared with low fluorescence intensity regions [15]. As the high fluorescence regions are mainly localized at bent structures, this suggests that folding of the nuclear lamina is associated with local accumulation of LMNA.

## 6. Discussion

**Figure 4:**
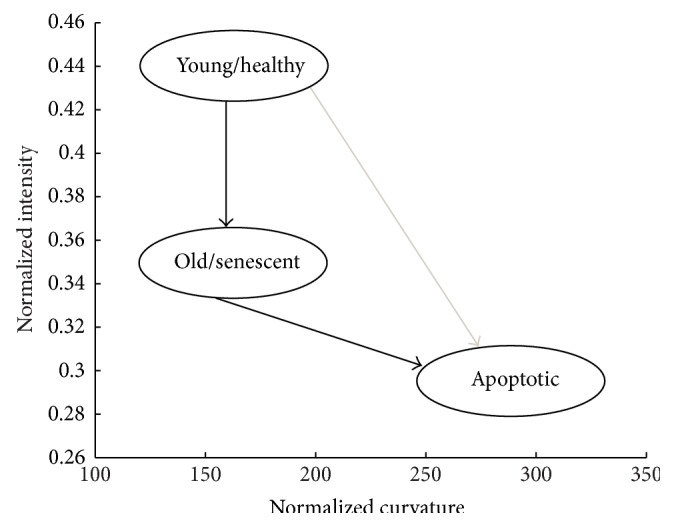
Normalized curvature and intensity measurements for a variety of cell populations are summarized. LMNA redistributes for aging and senescence, this is associated with a decrease in normalized intensity. Apoptotic cells undergo a severe morphological change, which is reflected in the increased curvature values. Apoptosis can be achieved either directly from healthy states or indirectly from old or senescent cells, adapted from [15].

Quantitative analyses of the nuclear lamina show that the mean normalized intensity of LMNA decreased from the young to the old and senescent cells. This indicates changes in the LMNA distribution in the nuclear lamina during aging and senescence. Changes in the average curvature between young cells and senescent and old cells are smaller compared to the intensity (Figure 4). The average curvature increases, however, significantly more in apoptotic cells compared to young or senescent cells [15]. Since cell apoptosis is increased in a population of senescent cells, this suggests that a major increase in folding of the nuclear lamina is secondary to an increase in local protein accumulation.

The nuclear lamins create a dynamic multifunctional platform combining both structural and regulatory functions. Regulatory functions of the nuclear lamins have been widely studied, and a dynamic multifunctional role is emerging [16]. Lamins directly bind to the chromatin, and chromatin recruitment of the nuclear envelope alters gene expression [24].

How changes in the structure of the lamina contribute to nuclear function in aging cells will be elucidated in future studies. Recent technical advances in monitoring and measuring the dynamics of the nuclear lamina structure [15, 24, 25] and lamina-chromatic dynamics on a single cell resolution [26] will open new opportunities to further understand how the structure of the nuclear lamina contributes to regulation of nuclear function.

It is not fully understood how the structure of the nuclear lamina changes. Based on the quantification of the biophysical features of the nuclear lamina, the following mechanical models are suggested: initially protein mobility decreases, leading to confined and localized LMNA accumulation in the nuclear envelop. This process is analogous to the entrapment of progerin proteins in the nuclear lamina. The local LMNA accumulation could result in a mechanical pressure that subsequently results in bending of the structure. Bending would start after the accumulation of the lamins reaches a critical threshold. When structural pressure of the nuclear lamina would be too large to maintain, the smooth ellipsoidal shape would deform, and the nuclear envelope structure would form an irregular shape. Advances in molecular imaging at the single cell level, in combination with high resolution studies of chromatin dynamics [26], enable the possibility to study how spatial changes of the nuclear lamina affect nuclear function in aging and senescent cells.
